# Firearm violence: a neglected “Global Health” issue

**DOI:** 10.1186/s12992-021-00771-8

**Published:** 2021-10-12

**Authors:** Meghan Werbick, Imran Bari, Nino Paichadze, Adnan A. Hyder

**Affiliations:** Center on Commercial Determinants of Health, Milken Institute School of Public Health, The George Washington University, 950 New Hampshire Ave, NW, Washington, DC, 20052 USA

**Keywords:** Firearms, Violence, Global Health, Guns, Intentional injury

## Abstract

Populations around the world are facing an increasing burden of firearm violence on mortality and disability. While firearm violence affects every country globally, the burden is significantly higher in many low- and middle-income countries. However, despite overwhelming statistics, there is a lack of research, reporting, and prioritization of firearm violence as a global public health issue, and when attention is given it is focused on high-income countries. This paper discusses the impact of firearm violence, the factors which shape such violence, and how it fits into global public health frameworks in order to illustrate how firearm violence is a global health issue which warrants evidence-based advocacy around the world.

## Background

Firearm violence is a pressing public health issue that is growing on a global scale. Mortality from firearms contributes more than 250,000 deaths each year worldwide, yet firearms still hold a massive commercial industry, with over 1 billion weapons in circulation as of 2017 [1, 2]. 857 million of these firearms are in civilian hands; an estimate that has increased by 32% from 2006 [1]. Even as research on non-communicable diseases and injuries (NCDIs) continues to progress, the burden of firearms on global mortality has received less attention. In fact, research, reporting, and prioritization of firearm violence is often concentrated in high- income countries (HICs), despite this issue affecting all populations globally.

Given the increasing importance of firearm violence, this commentary aims to restate the impact of firearms on health using selected research on the burden of firearm violence. It will demonstrate how politics, globalization, and spread of culture and ideals influence firearm violence on a global scale, and recognizing the diversity in definitions of the term “global health,” provides three definitions to demonstrate how firearm violence ought to be framed as a global health issue [3–5]. The need to recognize firearm violence as a global health issue is important to support prioritizing research and creating sound interventions, especially in low- and middle- income countries (LMICs).

### The impact of firearm violence

Injury and mortality rates from firearm violence, high-risk populations, causal factors, and societal issues which impact the rates of firearm violence are available from the Global Burden of Disease (GBD) data (Table 1). However, globally civilian populations share a majority of the burden of firearm violence; only 10% of mortality from firearms occurs in conflict situations [3, 6]. Mortality from firearms is also disproportionately concentrated in LMICs in South America, in addition to the United States; the GBD data from 2017 estimates that 50% of mortality from firearms occurred in countries that make up just 10% of the global population [1]. Worldwide, populations see similar patterns of firearm mortality; in every country, death rates are higher for men than women, and often the highest risk is among 20- to 24-year-olds. The majority of firearm deaths globally are in fact as a result of homicides [1].

**Table 1:** Mortality and Disability from Firearm Violence in 2017 (per 100,000)^a^

**Global Mortality and Disability**					
**Type of Violence**	**Prevalence**	**Mortality**	**YLDs**	**YLLs**	**DALYs**
Physical Violence	29.81	3.12	1.76	169.83	171.58
Self-Harm	1.56	0.83	0.08	33.36	33.44
Injury	122.38	0.56	5.86	27.05	32.91
**United States Mortality and Disability**					
Physical Violence	230.54	4.43	6.62	240.63	247.26
Self-Harm	3.0	7.69	0.07	294.17	294.24
Injury	223.55	0.27	3.71	12.11	15.82
**LMIC Mortality and Disability**					
Physical Violence	14.84	1.48	1.02	81.69	82.7
Self-Harm	1.25	0.54	0.07	25.28	25.34
Injury	82.08	0.42	4.50	22.17	26.67

^a^Naghavi M, Marczak LB, Kutz M, et al. Global mortality from firearms, 1990-2016. JAMA. 2018;320(8):792-814

*YLDs* Years lost due to disability, *YLLs* Years of life lost due to premature mortality, *DALYs* Disability adjusted life years.

Overall, fatality from firearm violence in the US has not decreased in over a decade. This is even as homicide risk is decreasing and suicide risk increasing over time; around 70% of homicides and 50% of suicides in the US involve firearms [7]. A disproportionate amount of these homicides are concentrated among black males; while white males have the highest risk for suicide by firearms, which also starts in younger populations and contributes to more than half of firearm mortality. At-risk populations also differ geographically in the US; suicide rates are 54% higher in rural areas while homicide rates are 90% higher in urban centers. Given these types of statistics, the US is an outlier in mortality from firearms as compared to other nations worldwide.

The GBD data separates firearm violence into three categories- deaths from firearms, unintentional injuries from firearms, and self-harm from firearms [1]. Collectively, mortality from these three categories contributes to over 250,000 preventable deaths per year, and over 46,000 Disability Adjusted Life Years (DALYs) lost (Table 1) [1]. Mortality from firearms is also significantly higher in LMICs; those in Central and South America, most notably Guatemala, Venezuela, and El Salvador, have mortality rates significantly higher at around 40 per 100,000 deaths as compared to the global average of 6 per 100,000 [1]. 2400 per 100,000 DALYs are lost from physical violence in these countries, compared to a global average of 171 per 100,000 [1].

Firearm violence also places a substantial burden on healthcare systems, economies, and societies around the world. In 2010 alone, the societal costs of firearm violence totaled $164 billion, the equivalent of 1.1% of the gross domestic product (GDP) of the United States in that same year. The 2015 Global Burden of Armed Violence report noted that almost USD 2 trillion could have been saved in a decade if the global homicide rate had been decreased from 7.4 to 3 deaths per 100,000; this is the equivalent of 2.64% of global GDP from 2010 [6].

### Factors that influence global firearm violence

The global political landscape directly influences firearm violence, particularly in LMICs [8]. The dynamic between high-income and low-income countries around the world also shapes the burden of firearm violence as policies, trade, and globalization worsen the problem. For example, extensive supply chains and the import of arms from HICs like the United States to other countries around the world highlight the importance of considering firearm violence as a *global* health issue [9]. More recently, the COVID-19 pandemic caused a surge of more than 90 million USD worth of firearms being exported to LMICs, particularly in Asia [9]. As scholars begin to better understand the private industry’s role in firearm violence, it is clear that such expansions in trade, supply chains, and marketing of arms on a global scale are contributing to the burden of firearm violence worldwide.

The global war on drugs has also extensively impacted the burden of firearm violence. In Mexico, government efforts to crack down on drug trafficking organizations resulted in an escalation of drug-related violence [10]. In addition, the political influence of HICs (such as the United States) has contributed to this issue, taking into account increasing drug consumption, loose firearms regulations, and regimes which are reportedly key actors in the drugs trade [8]. In fact, the political climate in HICs like the United States has the influence to shape how firearm regulations are interpreted, and enacted, in LMICs around the world. Most recently, the Mexican government has sued gun manufacturers in the United States for facilitating the trafficking of weapons with their negligent practices [11]. Roughly 70% of the trafficked firearms in Mexico come from the U.S., and 17,000 homicides can be linked to these weapons annually. The estimated damage of these trafficked weapons in Mexico is nearly 2% of the country’s GDP, which they will seek in the lawsuit, aiming to reduce further homicides in Mexico [11].

Additionally, globalization plays a role in increasing firearm violence as dynamics between HICs and LMICs change over time. Increases in foreign imports around the world have escalated competition within arms markets to produce higher performing weapons that can fire multiple types of ammunition, increasing both the accessibility and lethality of firearms [9]. Increasing globalization has also been found to promote the openness of trade and weaken public authority, making small arms ownership more likely [12]. The demand for small arms in HICs, which are responsible for the majority of the manufacturing and trading of these weapons, has caused the proliferation of small arms in LMICs as they are recycled down the “economic ladder.” [12] While research shows that attempting to limit globalization would not stop the trade of firearms, it is critical that the public health community more closely studies the movement of arms from HICs to LMICs [12].

Researchers have also suggested that “gun culture” from HICs (like the United States) has been “sold” to LMICs through various forms of media. In India, a country with historically low gun ownership rates, globalization has caused rates to increase [13]. In the city of Shivpuri, a program was sponsored which fast tracked the firearm permit process for men who were undergoing vasectomies, noting that this program allowed men to trade one aspect of their masculinity for another [13]. The shifting ideals surrounding the policies and accessibility of firearms caused by globalization has drastically impacted LMICs, a critical consideration for public health professionals as they begin to target this issue [12].

Finally, foreign policies can have an impact on arms trade and firearm violence in LMICs. In 2006, the United States was the only nation to dissent from the UN vote to implement stricter standards using an international arms trade treaty [14]. Traditionally the US policy positions on firearms have weakened efforts to further international agreements and gun control policies, and have particularly affected Latin America, as loopholes in laws have allowed for a consistent flow of arms across the US and Mexico border [14]. Alternatively, in the 1990s, the United States suspended arms exports to Paraguay until they were able to improve arms policies, illustrating how foreign policy can influence firearm violence in both positive and negative ways [14].

It is also clear that local policy can be successful in reducing violence; in 2021, Colombia introduced gun carrying restrictions in the cities of Bogotá and Medellín. Within 6 years, these cities saw a 22.3% reduction in firearm violence (adjusted for the standard annual reduction in control cities) [15]. Given this research, it is critical that global and local policy focuses their efforts on combating firearm violence.

### Firearm violence is a Global Health issue

As the body of research on firearm violence continues to grow, the public health and medical communities have shifted towards treating firearm violence not only as a preventable condition but also akin to an “infectious disease.” [16] Individuals who are more susceptible to firearm violence typically share various common exposures; like traditional disease epidemics. The environment, social networks, socioeconomic status, and education all influence the prevalence of firearm violence among communities and individuals [16].

This shift in knowledge has informed a variety of public health interventions aimed at decreasing the burden of firearm violence. However, to strengthen these interventions, it is critical that firearm violence be framed as a global health issue. As is evident from the factors which shape firearm violence, this issue takes place on a global scale, affecting the world’s most vulnerable populations. Politics, globalization, and the spread of culture and ideals through multi-media sources around the world has all contributed to the spread of firearm violence, which must be addressed by the global public health community [8, 12, 13].

Firearm violence also fits within the numerous frameworks and definitions proposed for ‘global health.’ In 2008, United Kingdom launched a 5-year strategy to target global health issues, defined as “*health issues where the determinants circumvent, undermine or are oblivious to the territorial boundaries of states, and are thus beyond the capacity of individual countries to address through domestic institutions*.” [3, 17] Firearm violence is certainly a global health issue by this standard, in urgent need of research, attention, resources, and intervention. It is also beyond the capacity of individual nations to address this through domestic institutions; many countries are struggling to achieve a social and health system in which firearm violence is no longer a cause for mortality, and due to the effects of globalization, it will take interventions in every country to curb the transfer of firearms between HICs and LMICs [12]. Koplan defines global health as “*an area for study, research, and practice that places a priority on improving health and achieving health equity for all people worldwide*.” [4] Reducing mortality from firearm violence is an area of critical research and practice and has direct health and equity implications. By better understanding patterns of violence in at-risk populations around the world and synthesizing current research on successful interventions, this knowledge can be used to develop strategies to reduce the burden of firearm violence on individuals, communities, and economies.

Scholars also state that for an issue to be considered a global priority, it should have four tenets: a global conceptualization of health, the synthesis of population-based approaches, the central concept of equity in health, and a cross-sectoral, interdisciplinary approach [5]. Firearm violence fits well within this conceptualization of global health; while all countries experience varying degrees of burden on their economies, healthcare systems, and populations as a result of firearm violence, globally it affects everyone and therefore warrants solutions rooted in research, evidence-based policy, and interventions. Given the applicability of firearm violence to the current definitions and frameworks for global health, the need for transnational solutions to firearm violence, and the ways in which firearm violence is influenced by global politics, it is apparent that given its vast impact on the health of world populations, firearm violence is a global health issue [8, 12, 13].

## Conclusions

Given the existing evidence of negative worldwide health outcomes associated with firearm violence, it is imperative that this becomes a priority topic of discussion in the field of global public health. As a result, this commentary argues that firearm violence needs to be increasingly framed as a major global health issue, further energize a wider global community of activists, and develop momentum around a narrative that is compelling in terms of health impact and known interventions. This pathway has done well for other global health priorities and firearm violence could follow the same success [18].

In order to achieve this change, a multi-disciplinary research effort surrounding firearms is necessary to create strong solutions. As such, we recommend three avenues of research that are of high priority in relation to firearm violence and its pertinence to global health. First, the health community should study the commercial determinants of health associated with firearm violence. Commercial determinants of health are defined by Kickbusch (2016) as “strategies and approaches used by the private sector to promote products and choices that are detrimental to health.” [19] Like many other industries, the firearms industry uses tactics such as marketing, lobbying, or corporate social responsibility to divert attention away from the negative health outcomes associated with their products- in this case, weapons [9]. Studying the issue through this perspective can provide a valuable perspective on one of the root causes of firearm violence and potential solutions.

Second, future research should be inclusive of both the direct and indirect health outcomes associated with firearm violence. Existing data has given the global public health community strong evidence of the direct burden of firearm violence on health. However, research is just beginning to understand how this violence can affect other aspects of health, such as mental and physical health outcomes associated with grief, fear, PTSD, costs of medical care, and other factors associated with firearm violence [20].

Finally, research must bring perspectives from LMICs to high-income countries, including those population groups most affected by firearms around the world. Studying the political economy, socio-cultural issues, and equity issues associated with firearm violence in different risk groups can help the global public health community best tailor policies and interventions to address this issue not just in the United States, but worldwide. Ultimately, bringing the best science to bear on this global perspective will help enable evidence-based advocacy to both national and international audiences to change mindset.

## Data Availability

Not applicable.

## Abbreviations

NCDIs: Non-Communicable Diseases and Injuries
LMICs: Low- and Middle- Income Countries
HICs: High Income Countries
GBD: Global Burden of Disease
YLDs: Years Lost due to Disability
YLLs: Years of Life Lost due to Premature Mortality
DALYs: Disability Adjusted Life Years
GDP: Gross Domestic Product
